# Improvement of Hypertension after Parathyroidectomy of Patients Suffering from Primary Hyperparathyroidism

**DOI:** 10.1155/2011/309068

**Published:** 2011-02-20

**Authors:** P. D. Broulik, A. Brouliková, S. Adámek, P. Libanský, J. Tvrdoň, K. Broulikova, J. Kubinyi

**Affiliations:** ^1^Third Department of Medicine, First Faculty of Medicine, Charles University in Prague, Prague, Czech Republic; ^2^Third Department of Surgery, First Faculty of Medicine, Charles University in Prague, Prague, Czech Republic; ^3^Department of Nuclear Medicine, First Faculty of Medicine, Charles University in Prague, Prague, Czech Republic

## Abstract

*Background*. Primary hyperparathyroidism (PHPT) is one of the most common endocrine conditions and is accompanied by hypertension and increased cardiovascular mortality. The purpose of this study was to evaluate the effect of parathyroidectomy on systolic and diastolic blood pressure (BP) in hypertensive patients with PHPT and whether hypertension occurs more frequently in PHPT than in control group. *Methods*. A total of 1020 patients with proved PHPT who underwent surgery were compared with with 1020 age, sex, BMI, and smoking status matched controls. We evaluated changes in serum calcium, parathyroid hormone (PTH), uric acid, and BP before and 6 months after surgery. *Results*. Parathyroidectomy corrected PHPT and resulted in a substantial fall in both mean systolic (150 ± 3.8 to 138 ± 3.6 mmHg) and mean diastolic pressures (97 ± 3 to 88 ± 2.8 mmHg) of the hypertensive subjects; *P* < .01. In these patients, PTH, calcium, and uric acid normalized. 726 patients from 1020 with PHPT (69.8%) were found to be hypertensive whilst only 489 (47.8%) from 1020 of our control group. *Conclusion*. Parathyroidectomy in hypertensive patients reduces systolic and diastolic BP. PHPT is accompanied by a variety of metabolic complications, which are a risk factor for hypertension, and parathyroidectomy can improve these metabolic complications.

## 1. Introduction

Primary hyperparathyroidism (PHPT) is one of the most common endocrine conditions particularly in postmenopausal women. PHPT is being diagnosed with increasing frequency mainly as a result of the introduction of routine serum calcium measurements. This practice has also led to a different mode of presentation of PHPT. Earlier PHPT was considered a rare illness characterized by bone disease and urinary calculi. Many new clinical features, such as hypertension, mental disturbances, myopathy, peptic ulcer disease, pancreatitis, and cholelithiasis have been added to the clinical spectrum of PHPT [1].

It has been reported that patients suffering from symptomatic PHPT have increased mortality before and after parathyroidectomy [2]. This increase in mortality seems to be mainly due to an overrepresentation of cardiovascular death [3]. Hyperparathyroidism may coincide with or induce hypertension [4].

Hypertension is common in hyperparathyroidism but the cause of this association is not well recognized. It was first studied systematically by Hellstrom et al. [5]. Since then various authors reported hypertension to be present in between 10% and 70% of patients with primary hyperparathyroidism [6]. The wide variation in the prevalence may be due to differences in methods of blood pressure measurement, diagnostic criteria for hypertension, and severity of hyperparathyroidism. Main sources of variability are related to the observer, the environment, and the device. Some studies show cure or improvement in a proportion of patiens [7, 8] but others report no significant changes in blood pressure after surgery [9, 10]. The present study is a comparison study of the blood pressures of a large group of patients with PHPT and of patients receiving health examination at outpatients ward. We wished to determine whether hypertension occurs more frequently in PHPT than in control group and whether the change in blood pressure after surgery is significant.

## 2. Material and Methods

### 2.1. Study Subjects

The whole series comprise 1020 patients (847 female and 173 males aged 20–76) with biochemically, surgically, and histologically proved PHPT during a period of 9 years (1998–2007) at Third Internal Clinic Medical School of Charles University of Prague

The diagnosis of PHPT was confirmed by histopathological diagnosis based on combination of different variables, including size and histological pattern of the excised glands. Off the 1020 patients who underwent parathyroidectomy 960 had an adenoma (chief cells) and 60 hyperplasia. None of the 1020 patients with PHPT had multiple endocrine neoplasia. 

Previous antihypertensive treatment was documented in 64% of all PHPT patients with hypertension. Antihypertensive use in hyperparathyroid patients with hypertension was ACE inhibitors (52%), beta blockers (24%), diuretics 25%, and calcium channel blockers (29%). All patients were suffering from essential hypertension stage I or II.

The control group was drawn from 2020 patients receiving periodic health examination at outpatient ward in Third Medical Department of Prague Charles University Medical School from January 2004 to December 2005. The control group was carefully selected so that each control was matched with an individual patient with regard to sex, similar age, BMI, and smoking status. The following exclusion criteria were used: impaired renal function, malignant and secondary hypertension, and myocardial infarction. All patients of the control group have been normocalcemic and with normal iPTH. For comparison also were used published data on the population prevalence awareness and control of hypertension in the Czech population from 1985 to 2001 [11].

**Study Protocol**

The standardized baseline screening included complete history, physical examination, skeletal radiographs, sonography of the abdomen, electrocardiogram, and DEXA. Serum concentrations of calcium, uric acid, and creatinine were determined by standard laboratory techniques in the same serum specimen in each patient. (Technicon Autoanalyzer Roche). Normal reference values were calcium 2.15–2.65 mmol/L, creatinine 44–105 umol/L, and uric acid 140–340 umol/L. The biochemical criteria for renal functional impairment consisted of a level of serum creatinine greater than 110 umol/L. Serum intact PTH was assayed by a two-site immunochemiluminometric assay (Bayer Corp Chiron, Halstead UK) with mean values 1.6–6.9 pmol/L.

X-ray films were taken of the hands, skull, and kidneys to look for bone disease and renal calculi or nephrocalcinosis. Also ultrasound examinations were taken of the kidneys and gallbladder to look for calculi.

The examination consisted of a physician completed questionnaire, which included questions of whether at any time in the past the patient had received treatment for high blood pressure (BP) or had hypertension diagnosed and whether they were currently taking antihypertensive medication. The names of currently prescribed medications were recorded and validated against medication containers when possible.

Height and body weight were measured with participants standing without shoes and heavy outer garments. Body mass index (BMI) was calculated as weight divided by height squared (kg/m^2^) as a measure of relative weight. Obesity was defined as BMI > 30 kg/m^2^ for both sexes.

Smoking was classified as current, ex-(not for at least 1 year), and nonsmoker.

BP was measured three times with the patients in sitting position following the recommendations of the current European guidelines for blood pressure measurement. The average of the three measurements was taken as the reference values for each patients [12].

In this study hypertension was defined as a mean systolic BP (SBP) > 140 mmHg and or a mean diastolic BP (DBP) > 90 mmHg or current treatment with antihypertensive drugs. Treatment of hypertension was defined as current use of a prescribed medication affecting BP. Blood pressure in PHPT patients and controls was measured on the right arm with subject in the sitting position after at least 5 minutes at rest by standard mercury sphygmomanometers, and correctly sized cuffs were used.

To withdraw antihypertensive treatment before the examination was considered hazardous. Antihypertensive drugs and doses were not changed throughout the study. None of the PHPT patients complained from recent angina pectoris, dyspnea, dizziness, or syncope.

Follow-up examinations 6 months after parathyroidectomy included questionnaire, physical examination, the same blood chemistries as before operation, and blood pressure measurement.

The study was done in accordance with the principles of the Helsinki declaration, and the procedures followed were in accordance with institutional guidelines. The study was approved by the Hospital ethics committee, and all patients gave written informed consent.

Statistical and discriminant analyses of the laboratory tests were performed by analysis of variance followed by the multiple range test [13]. All mean values are presented with one standard deviation (SD). A *P* value less than  .05 (two sided test) was accepted as level of significance. The difference in percentages in both groups was tested using chi-square test. The differences between the preoperative and postoperative mean values were evaluated using the paired or unpaired Student's *t*-test.

## 3. Results

Parathyroidectomy corrected hyperparathyroidism in all cases. The serum levels of calcium, iPTH and uric acid normalized after parathyroidectomy (Table 2).

The changes of blood pressure following surgical correction of PHPT were based on blood pressure recordings 6-7 months postoperatively. The decrease in blood pressure was seen not only in individuals never receiving antihypertensive therapy but also in individuals on medication for hypertension. Parathyroidectomy resulted in a substantial fall in both mean systolic (150 ± 3.8 to 138 ± 3.6 mmHg) (*P* < .01) and mean diastolic pressures (97 ± 3 to 88 ± 2.8 mmHg) (*P* < .01) of the hypertensive subjects (Tables 2 and 3). We did not observe the decrease of BP in PHPT patients without hypertension. The mean systolic (*P* < .01) and diastolic BP (*P* < .05) decreased also in hypertensive patiens on antihypertensive therapy (Table 3).

Hypertension was documented preoperatively in 726 patients with PHPT (69.8%) whilst 294 of the patients were normotensive. The blood pressure recorded in 726 subjects with hypertension averaged 150 ± 3.8 mmHg (systolic) and 97.0 ± 3.0 (diastolic) in women and in men. There were 610 women and 116 men (Table 1). In the control group 489 patients of the entire group of 1020 patients (47.9%) had hypertension whilst 531 of the patients were normotensive. There were 410 women and 79 men (Table 1). Parathyroid adenomas occurred with similar frequences in the hypertensive and normotensive patients.

In the patients with PHPT no one had severe hypertension, diastolic pressure over 120 mmHg. Hypertension appeared to be significantly more common in the PHPT (72.1% in the case of women and 67.4% in the case of men) in comparison with our sex, age, BMI, and smoking status matched control group (women 45.9% and men 49.9%) (*P* < .001). The estimated percentage of hypertensive patients with PHPT receiving pharmacological treatment was 64.5%. It was slightly higher in females. 

There is not statistically significant difference between BMI of all patients with PHPT and our BMI-matched control group (30.4 ± 2.6 kg/m^2^ versus 29.3 ± 3.2 kg/m^2^) (*P* > .05). However there is relationship between BMI and hypertension inside the group of patients suffering from PHPT. Average BMI index for women and men with PHPT and hypertension was (32.8 ± 2.4 kg/m^2^) and in the present study is higher than BMI index for women and men with PHPT and without hypertension (29.2 ± 2.04) (*P* < .01) Table 4. Hypertension in hyperparathyroid patients was significantly associated with higher body mass index. After surgery there was a significant decrease in blood pressure however we did not see significant effect on BMI (Table 2).

Patients with PHPT before operation had increased iPTH and serum calcium in comparison with control subjects. Statistically significant differences between normotensive and hypertensive patients with PHPT were evident preoperatively with respect to the serum iPTH and uric acid (*P* <  .01) (Table 4). PTH and calcium concentration normalized six months after parathyroidectomy and were not different from controls at the follow-up visit (Table 2). Preoperative serum calcium levels were not significantly higher in the hypertensive than in the normotensive patients with PHPT. No correlation could be found between serum calcium levels and systolic and diastolic blood pressure between patients with PHPT and hypertension and PHPT without hypertension before surgery. Serum uric acid levels were at baseline significantly higher in patients with PHPT when compared to controls and were similar in the hypertensive and normotensive patients with PHPT (Table 1) There were significant differences in mean serum uric acid levels between the whole group with PHPT (368 ± 54 umol/L) and the whole control group (266 ± 49 umol/L); *P* < .01. Also significant differences were between the group normotensive (344 ± 42 umol/L) and hypertensive (395 ± 58 umol/L) patients with PHPT; *P* < .01. Six months after parathyroidectomy serum uric acid fell significantly in hypertensive from 395 ± 58 to 290 ± 56 umol/L in patients with PHPT (*P* < .01) (Table 2).

Immunoreactive parathormone was higher in the hypertensive patients with PHPT (21.6 ± 4.2 pmol/L) than in the normotensive patients with PHPT (12.2 ± 2.8 pmol/L) (*P* < .01) (Table 4).

Kidney involvement due either to deposition of calcium in the renal parenchyma or to nephrolithiasis was present in 31.4% of our patients with PHPT and only 6% in control group. The frequency of renal calculi was similar in patients with PHPT and hypertension (32%) and patients with PHPT without hypertension (29%). Preoperative serum creatinine levels were statistically higher in the hypertensive patients (93 ± 6 umol/L) than normotensive ones with PHPT (74 ± 6 umol/L) and changed in the case of hypertensive patients significantly after operation (71 ± 8 umol/L) (*P* < .01), (Table 1). The preoperative SBP was not significantly correlated to serum creatinine. The patients suffering from chronic renal failure at the time of surgery, creatinine more than 120 umol/L were excluded from our analysis. 

Adjustment for smoking status did not alter the results. Number of smoking patients with PHPT (16.5%) was similar to the matched control group (17.5%).

## 4. Discussion

In this retrospective study, we have demonstrated that parathyroidectomy reduces blood pressure in hypertensive patiens with PHPT. The decrease in blood pressure was seen not only in individuals never receiving antihypertensive therapy but also in individuals on medication for hypertension indicating the pathogenetic participation of PHPT on blood elevation. 

In the present large series of patients with clinically and biochemically proven primary hyperparathyrodism we found that 69.8% of patients have been suffering from hypertension. It has been suggested that arterial hypertension is common in PHPT, and our date confirm, this hypothesis. The 69.8% prevalence of hypertension between patients with PHPT is higher as compared with 47% prevalance of hypertension in general population in Czech Republic [11] and also from our sex, age, BMI control group (48%). The mean age of all our patients with PHPT at the time of diagnosis is 58.8 years which is almost the same like in general population (60 years) where prevalence of hypertension is 47% [14].

Several pathogenetic mechanisms could be responsible for higher prevalence of hypertension in PHPT. Suggested mediators have included hypercalcemia, high PTH, plasma renin, renal functions, uric acid, and BMI. Clinical and experimental evidence has suggested a relation between the serum calcium level and blood pressure. Hypercalcemia induced in man by a variety of ways including administration of vitamin D or abrupt intravenous infusion of calcium led to acute elevation of blood pressure and its prompt return to normal values when normal levels of serum calcium are restored [15]. A close correlation has been demonstrated between parathyroid hormone and intracellular calcium, indicating that PTH may act as an ionophore for calcium entry into the cells. The correction of the hyperparathyroid condition normalizes intracellular calcium concentration [16].  We did not find any correlation between serum calcium level and hypertension in patients suffering from PHPT.

Raised concentration of parathormone can be responsible for the hypertension. PTH was higher in our hypertensive patients with PHPT than in the normotensive patients with PHPT. However fall in hormone concentrations after surgery does not correspond to a fall in blood pressure in all our patients. However indirect effect of PTH on blood pressure cannot be ruled out. Results of  [15] suggest that hypertension associated with clinical PHPT results from either direct or indirect effects of PTH excess. Some studies have shown that patients with essential hypertension have a higher serum concentration of PTH than normotensive individuals [17]. 

The administration of parathyroid hormone stimulates the secretion of renin in dogs and in humans and is associated with an increase in plasma renin activity [18]. We have also shown raised plasma renin activity in healthy subjects without hypertension after administration of PTH [19]. Also the chronic elevation of parathormone in PHPT was accompanied by high plasma renin activity. After parathyroidectomy plasma renin activity levels fell to normal. The most plausible explanation may be the renal vasodilatory effect of PTH. A decrease in stretch of the afferent arterioles stimulates renin release from the juxtaglomerular apparatus [20]. 

There is a possibility that hypercalcemia and/or a chronic exposure to high PTH levels might affect the arterial tone. The patients with PHPT exhibit an impaired endothelial vasodilatory function and parathyroidectomy can reverse this defect [21].

Recent experimental and clinical evidence supports the possibility that an elevated uric acid level may lead to hypertension [22]. Hyperuricemia carries an increased relative risk for hypertension developing within 5 years, independent of other risk factors. Both hyperuricemia and gout occur with increased frequency in hyperparathyroidism, and PHPT causes a reduced clearance of uric acid and raises the serum uric levels [23]. In our patients serum uric acid was higher in the PHPT patients than in control subjects. There were significant differences in mean serum uric acid level between the group with PHPT normotensive and hypertensive. After parathyroidectomy serum uric acid level fell significantly in hypertensive patients with PHPT.

PHPT is known to cause renal damage, and this might be blamed for the raised blood pressure [24]. From our data it does not seem very convincing that renal damage or renal calculi are playing an important role in etiology of hypertension in PHPT. The mean serum creatinine levels in the hypertensive and normotensive patients with PHPT were in normal range indicating that the hypertension of hyperparathyroidism is not the consequence of advanced renal parenchymal damage. There was no difference in the frequency of renal calculi between normotensive and hypertensive hyperparathyroid patients.

We did not find statistically significant changes in BMI between patients with PHPT and control group. However patients with PHPT and hypertension are heavily reporting BMI than patients with PHPT without hypertension. Increased body weight may contribute to the reported association between PHPT and hypertension. After operation there was a significant decrease in blood pressure; however we did not see significant effect on BMI. Vitamin D is fat soluble and is sequestered by adipose tissue. Thus increased body weight may promote vitamin D deficiency, resulting in secondary hyperparathyroidism. Secondary hyperparathyroidism appears to increase the risk of developing parathyroid adenoma [25]. After adjusting for smoking status we did not observe any connection between smoking and hypertension in our patiens with PHPT.

There is increasing evidence that PHPT is associated with an increased risk of cardiovascular event [26]. Patients with PHPT have been reported to have a greater prevalence of cardiovascular abnormalities than the normal population [27]. Patients with PHPT seem to have an increase in mortality, and this seems mainly due to an overrepresentation of cardiovascular death. PHPT is reported to be associated with hypertension, disturbances in the renin-angiotensin-aldosterone system, cardiac arrhythmias as well as structural and functional alterations in the vascular wall [28].

In conclusion PHPT is a common disorder often associated with hypertension. Parathyroidectomy resulted in a substantial fall in both SBP and DBP in hypertensive patients. From our work it is evident that PHPT is accompanied by a variety of metabolic complications which are a risk factors for hypertension and that parathyroidectomy can improve these metabolic complications. Our data support the recommendation that parathyroidectomy should be performed in all patients with PHPT because the procedure stops the progression of hypertension. The present data also reveal new possibilities in the field of pathogenetic mechanism of hypertension in PHPT. The present study strengthens the view that cardiovascular derangements are important indications for surgery in PHPT.

Our results support the hypothesis that untreated hyperparathyroidism is a risk factor for hypertension. Reducing blood pressure in patients with PHPT may be beneficial for long-term cardiovascular health. 

## Figures and Tables

**Table 1:** Preoperative demographic, biochemical data and BMI in patients with primary hyperparathyroidism and controls. Results are presented as mean values ± SD. ^●^ *P* < 0.01—patients with primary hyperparathyroidism versus controls.

	No.	Age (yrs)	Gender Female/male	iPTH pmol/L	Uric acid mmol/L	BMI kg/m^2^	Serum creatinine umol/L	Serum calcium mmol/L
All patients with PHPT	1020	58 ± 14	F: 847 M: 173	16.9 ± 2.8^●^	368 ± 45^●^	31.4 ± 2.6	74 ± 5	3.10 ± 0.1^●^
Patients with PHPT with hypertension.	726	59 ± 15	F: 610 M: 116	21.6 ± 4.2^●^	395 ± 58^●^	32.8 ± 2.5	93 ± 3.3	3.01 ± 0.3^●^
All control patients	1020	60 ± 15	F: 847 M: 173	4.7 ± 0.6	266 ± 42	29.3 ± 3.0	71 ± 9	2.40 ± 0.3
Patients with hypertension in control group.	489	61 ± 14	F: 410 M: 79	4.6 ± 1.2	290 ± 56	30.9 ± 3.2	73 ± 4	2.42 ± 0.2

**Table 2:** Preoperative and postoperative biochemical data, BMI, and arterial blood pressure in hypertensive patients with primary hyperparathyroidism. Results are presented as mean values ± SD.

	Preoperatively *n* = 726	Postoperatively *n* = 726	*P* value
PTH (pmol/L)	20.6 ± 8.2	5.4 ± 1.5	*P* < .01
Uric acid (mmol/L)	395 ± 58	290 ± 56	*P* < .01
BMI (kg/m^2^)	32.8 ± 2.5	33.2 ± 3.0	*P* > .05
Calcium (mmol/L)	3.01 ± 0.1	2.31 ± 0.3	*P* < .01
Systolic BP mmHg	150 ± 3.8	138 ± 3.6	*P* < .01
Diastolic BP mmHg	97 ± 3	88 ± 2.8	*P* < .01

**Table 3:** Preoperative and post-operative biochemical data, BMI and arterial blood pressure in hypertensive patients with primary hyperparathyroidism on antihypertensive medication. Results are presented as mean values ± SD.

	Preoperatively *n* = 663	Postoperatively *n* = 663	*P* value
PTH (pmol/L)	21.0 ± 7.2	5.6 ± 1.5	*P* < .01
Uric acid (mmol/L)	344 ± 45	265 ± 46	*P* < .01
BMI (kg/m^2^)	32.8 ± 2.5	33.1 ± 3.0	*P* > .05
Calcium (mmol/L)	3.0 ± 0.1	2.31 ± 0.3	*P* < .01
Systolic BP mmHg	135 ± 5.0	125 ± 3.0	*P* < .01
Diastolic BP mmHg	85 ± 3	81 ± 2.8	*P* < .05

**Table 4:** Comparison of biochemical data and BMI in hypertensive patients with PHPT and normotensive patients with PHPT. Results are presented as mean values ± SD. ^●^ *P* < .01—patients with PHPT and hypertension versus patients with PHPT without hypertension.

	PHPT with hypertension *n* = 726	PHPT without hypertension *n* = 294
s.Calcium mmol/L	3.01 ± 2.4	3.1 ± 2.04
iPTH pmol/L	21.6 ± 4.2**^●^**	12.2 ± 2.8
Uric acid mmol/L	395 ± 58**^●^**	344 ± 42
BMI kg/m^2^	32.8 ± 2.4**^●^**	29.0 ± 2.04
